# Effective Population Size, Gene Flow, and Species Status in a Narrow Endemic Sunflower, *Helianthus neglectus*, Compared to Its Widespread Sister Species, *H. petiolaris*

**DOI:** 10.3390/ijms11020492

**Published:** 2010-02-02

**Authors:** Andrew R. Raduski, Loren H. Rieseberg, Jared L. Strasburg

**Affiliations:** 1 Department of Biology, Indiana University, Bloomington, IN 47405, USA; E-Mail: aradus2@uic.edu; 2 Department of Botany, University of British Columbia, Vancouver, B.C. V6T 1Z4, Canada; E-Mail: lriesebe@interchange.ubc.ca

**Keywords:** gene flow, species delimitation, genetic variation, reproductive barriers, endemism

## Abstract

Species delimitation has long been a difficult and controversial process, and different operational criteria often lead to different results. In particular, investigators using phenotypic *vs.* molecular data to delineate species may recognize different boundaries, especially if morphologically or ecologically differentiated populations have only recently diverged. Here we examine the genetic relationship between the widespread sunflower species *Helianthus petiolaris* and its narrowly distributed sand dune endemic sister species *H. neglectus* using sequence data from nine nuclear loci. The two species were initially described as distinct based on a number of minor morphological differences, somewhat different ecological tolerances, and at least one chromosomal rearrangement distinguishing them; but detailed molecular data has not been available until now. We find that, consistent with previous work, *H. petiolaris* is exceptionally genetically diverse. Surprisingly, *H. neglectus* harbors very similar levels of genetic diversity (average diversity across loci is actually slightly *higher* in *H. neglectus*). It is extremely unlikely that such a geographically restricted species could maintain these levels of genetic variation in isolation. In addition, the two species show very little evidence of any genetic divergence, and estimates of interspecific gene flow are comparable to gene flow estimates among regions within *H. petiolaris*. These results indicate that *H. petiolaris* and *H. neglectus* likely do not represent two distinct, isolated gene pools; *H. neglectus* is probably more accurately thought of as a geographically restricted, morphologically and ecologically distinct subspecies of *H. petiolaris* rather than a separate species.

## 1. Introduction

Species are one of the fundamental units of biological organization, and species delimitation is a major goal in evolutionary biology [1]. A wide range of different data types and methodological approaches has been used to delimit species, and often different data or approaches lead to dramatically different outcomes [2,3]. In the absence of genetic and molecular data, many species were initially described based on morphological, ecological, or life history criteria. When such traditionally delimited species are examined using molecular data, in some cases deeply divergent lineages have been identified within species showing little or no phenotypic variation [4]; in other cases the reverse is true – phenotypically divergent populations appear to form a single gene pool [5]. In plant systems in particular, different individuals or populations may appear phenotypically diverged due to phenotypic plasticity or local adaptation, while still experiencing high levels of gene flow and little genetic differentiation throughout most of the genome [6–8].

The annual sunflower *Helianthus neglectus* has a restricted distribution on sand dunes in southwestern Texas and southeastern New Mexico. *Helianthus neglectus* is phenotypically very similar to its much more widespread sister species, the prairie sunflower, *H. petiolaris,* which is found throughout much of the Great Plains and western United States. There are limited morphological differences, including some differentiation in head size as well as leaf size and structure. The two species also display edaphic differences, with *H. neglectus* limited to deep sand dunes, while *H. petiolaris* is most commonly found in sandy soils, but not exclusively so [9]. While *H. neglectus* has a much more restricted range and generally occurs in less stable, more sandy soils, the species’ habitats can often be found adjacent to each other without a clear geographic barrier separating the ranges. Natural hybrids between the two species are not uncommon where their ranges overlap [9,10], although the degree to which introgression occurs between them remains unexamined.

Previous cytological and molecular research has confirmed a close relationship between the two species. Allozyme and restriction fragment analysis suggests that the *H. neglectus* gene pool consists largely of a subset of *H. petiolaris* alleles [11]. The two species differ by one or two reciprocal translocations that create a chromosomal sterility barrier to some degree [12,13]. However, they display F1 pollen viability levels of 75–80%–substantially higher than almost all other annual sunflower interspecies crosses [12,13], and not much lower than some intraspecific crosses [14]. Although classified as distinct species, in his initial description of *H. neglectus*, Heiser [13] described the decision of whether or not *H. neglectus* should be considered a distinct species or a subspecies of *H. petiolaris* as “somewhat arbitrary”.

Relationships between *H. neglectus* and *H. petiolaris* have not been addressed in detail for two decades [15], during which time the increased availability of multi-locus sequence datasets and methodological advances have made much more precise demographic inferences possible. In this study, we examine the patterns of genetic diversity and divergence in these two species using sequence data from nine loci derived from an expressed sequence tag (EST) database for various *Helianthus* species. We find that the two species harbor comparable levels of genetic diversity—far more diversity in *H. neglectus* than is generally realistic for an isolated species of its limited geographic distribution and population size—and display virtually no genetic divergence. In addition, estimates of gene flow between the two species are similar to estimates among geographic regions within *H. petiolaris*, and the overall geographic distribution of genetic variation in both species together reflects a pattern of isolation by distance in a single large gene pool, rather than one of two distinct gene pools. We consider these results in the context of distributional differences between these two species as well as their species status.

## 2. Results and Discussion

### 2.1. Genetic Diversity and Differentiation

Sequence data from nine nuclear loci was obtained through direct sequencing and molecular cloning techniques. Overall aligned sequence length ranged from 454 to 1,326 bp, with an average size of 725 bp (6,525 bp total). Basic summary statistics are given in Table 1. Average per-bp silent π and θ values, respectively, were 0.036 and 0.051 for *H. petiolaris*, and 0.041 and 0.048 for *H. neglectus*. The large majority of the genetic variation is distributed within species with very little divergence between species, as indicated by the average net divergence across loci, which was 0.14% (range 0–0.46%). A summary of the four types of substitutions described in Wakeley and Hey [16] are given in Table 2. These substitutions are the number of sites polymorphic in each species, in both species, and the number of differences that are fixed between species. The degree of variation within each species is again showcased using these statistics, with an average of 45 and 23 polymorphisms unique to *H. petiolaris* and *H. neglectus,* respectively (based on pairwise sequence diversity levels within each species, the difference between species in the number of unique polymorphisms reflects sample size differences rather than overall genetic diversity differences). Surprisingly, none of the 847 polymorphisms in the overall dataset is distributed as a fixed difference between *H. petiolaris* and *H. neglectus*.

In AMOVAs, an average of 70.7% of variation was found within regions (P values for all nine loci <0.0001), compared to 25.9% variation found among regions (P values across loci range from <0.0001 to 0.0031) within species and 3.4% variation found between species (P values range across loci from 0.001 to 0.504; three of nine loci significant at the 0.05 level). In neighbor joining trees using *Bahiopsis lanata* and *B. reticulata* as outgroups, *H. petiolaris* and *H. neglectus* haplotypes had a polyphyletic relationship at all nine loci. Neighbor joining trees and 50% majority-rule bootstrap consensus neighbor joining trees for all nine loci are given in Supplementary File S1.

Results for Tajima’s D and Fu’s Fs tests are given in Table 3. In *H. petiolaris*, two loci were significantly negative using Tajima’s test, and all nine loci were significant using Fu’s test. The number of significant results for Tajima and Fu’s tests decreased to zero and eight, respectively, after Bonferroni correction. For *H. neglectus,* no loci were significant for Tajima’s test, but seven loci were significant for Fu’s test (four after Bonferroni correction). Fs is considered more sensitive to population size change, with negative values indicating population growth, while D is considered more sensitive to selection [17]. Given that, our results are most consistent with selective neutrality at all loci and population growth in both species. HKA tests were not significant across all loci, also indicating selective neutrality.

Mantel tests were performed on *H. petiolaris* populations and on all populations of both species together. Correlations between geographic and genetic distance averaged across loci are very low and non-significant both for *H. petiolaris* individually and for both species together (0.004 and 0.028, respectively). No individual locus shows a significant positive correlation for *H. petiolaris* individually, and a single locus, JLS2899, is significant for both species together.

### 2.2. Effective Population Sizes and Gene Flow Rates

Estimates of effective population sizes and long-term gene flow rates made using MIGRATE are given in Tables 4 and 5, respectively. Modal population size estimates for the three *H. petiolaris* regions range from 240,000 to 640,000. In contrast, the modal population size estimate for *H. neglectus* is roughly 2.3 million; the low end of the 95% confidence interval is close to one million. This estimate is somewhat smaller than a previous estimate of the *H. petiolaris* species-wide effective size [18], but it is still far larger than would be expected for an isolated species with the geographic range and census size of *H. neglectus*.

Likewise, gene flow estimates are not consistent with increased isolation between *H. neglectus* and *H. petiolaris* relative to isolation among regions within *H. petiolaris*. Modal gene flow estimates are N_e_m = 0.01 (the smallest value possible based on bin size), although confidence intervals are quite wide, with a lower bound of zero in all cases and upper bounds ranging from 3.8 to 5.5. Posterior distributions of gene flow estimates between *H. neglectus* and various *H. petiolaris* groups are broadly overlapping with posteriors for gene flow within *H. petiolaris*. When the six gene flow estimates involving *H. neglectus* are compared to the six estimates within *H. petiolaris*, the former have slightly higher average confidence interval upper bounds (4.57 *vs.* 4.29), median values (1.55 *vs.* 1.49) and mean values (0.96 *vs.* 0.78). In addition, although the confidence intervals are broadly overlapping, confidence interval upper bounds, median values, and mean values are slightly higher for interspecific comparisons involving the sympatric *H. petiolaris* region *vs.* interspecific comparisons involving the allopatric (eastern and western) *H. petiolaris* regions. This pattern is expected if the two species are exchanging genes in sympatry.

### 2.3. Species Status of H. neglectus

Defining distinct species has long been a goal of evolutionary biologists as well as natural historians. Although a modern consensus definition of a species is still a topic of debate [19,20], operational methods of identifying species have historically utilized morphological or ecological traits in addition to crossing data. Investigators have incorporated cytological and molecular data as it has become available, and the value of examining multiple sources of data is generally recognized [1,21,22]. On occasion, the addition of molecular data has led to substantial reevaluation of species boundaries and relationships [e.g., 23,24].

Population genetic theory predicts that species with restricted ranges (and presumably concomitant small population sizes) should have less genetic variation than their widespread counterparts. This is expected mainly due to the increased importance of genetic drift in small populations [25]. When comparing the narrowly distributed sunflower species *H. neglectus* with its widespread sister species *H. petiolaris*, we do not observe this pattern at any of the nine loci examined here. Because of the vast differences in species’ ranges and census population sizes, we expected far more genetic variation in *H. petiolaris*. However, levels of genetic variation in *H. neglectus* were comparable to (and in some cases *higher* than) those in *H. petiolaris.* In addition we found very little genetic divergence between the two species, as well as comparable levels of interspecific and intraspecific gene flow.

Three possible scenarios may explain the surprisingly high levels of genetic variation in *H. neglectus*. If *H. neglectus* is the product of a recent split from the more widespread *H. petiolaris* and *H. neglectus* populations are currently still undergoing lineage sorting, polyphyletic relationships among species may be expected. However given the restricted range of *H. neglectus* it is unlikely that the species would be able to sustain such high levels of variation that are observed for a significant length of time. Similarly, if *H. neglectus* was much more widespread than it is at present, high levels of variation may still be found in the much smaller species range. However, there is no evidence that this is the case. The values of Fu’s Fs and Tajima’s D presented here are more consistent with recent *H. neglectus* population growth rather than decline, making this scenario unlikely as well.

A more plausible explanation for the observed pattern is high levels of ongoing gene flow between the two nominal species. In his initial description of *H. neglectus*, Heiser [13] reported artificial hybrids with varying degrees of fertility (measured by pollen staining viability of 9–80%). In addition, apparent hybrids are not uncommon in areas of range overlap [9,10]. A previous study examining pollen viability in hybrids between *H. annuus* and *H. petiolaris* show drastically lower levels of viability [average 4.8%–26]. Despite these low F1 pollen viability levels, recent studies have shown evidence of long-term, ongoing introgression between *H. annuus* and *H. petiolaris* in both directions [18,27]. Given the relatively higher F1 pollen viabilities between *H. neglectus* and *H. petiolaris*, pollen sterility is unlikely to prevent significant introgression in sympatry.

Our modal gene flow estimates from MIGRATE among all regions in both species are surprisingly low, although confidence intervals are very broad and encompass very high gene flow levels as well (Table 5). These results are inconsistent with higher estimates of gene flow within *H. petiolaris* (N_e_m ∼ 1) previously reported [28–30]. The Bayesian implementation of MIGRATE can have a downward bias in gene flow estimates for a wide range of θ values, including the range of values estimated here [31]; this may explain the discrepancy we see between estimates made using the different methods. However, we see no obvious reason why intraspecific estimates would be more strongly biased than interspecific estimates, so we do not expect the relative levels of interspecific *vs.* intraspecific gene flow to be affected. The Bayesian implementation of MIGRATE estimates effective sizes well [substantially better than the maximum likelihood implementation–31], suggesting that our estimate of *H. neglectus*’ effective size is reliable; as mentioned above this estimate is not consistent with an isolated species with a very small geographic range and census size, except under very unrealistic demographic conditions.

Within *H. petiolaris* there are two recognized subspecies, ssp. *petiolaris* and ssp. *fallax* [13], distinguished by some minor phenotypic differences as well as at least one chromosomal rearrangement; in addition, there is some cytological variation within ssp. *petiolaris* [32]. Generally, ssp. *petiolaris* is found throughout the Great Plains up to the Rocky Mountains, while ssp. *fallax* is found in the southwestern United States [9]; the range of *H. neglectus* overlaps or nearly overlaps with each *H. petiolaris* subspecies. Crosses between cytological races of *H. petiolaris* show varying degrees of fertility loss relative to crosses within races [32]; interestingly, hybrids between *H. neglectus* and both subspecies of *H. petiolaris* show pollen viabilities within the range of variation seen in crosses between the *H. petiolaris* subspecies and cytological races [9,13,32].

Based on the comparable levels of genetic variation in both species and similar inter- and intraspecific gene flow and effective population size estimates from MIGRATE, the validity of *H. neglectus*’ distinctness as a species should be more closely examined. Both species examined show high levels of genetic variation, which is in accordance with previous studies of *Helianthus* species [18]. Such high levels of variation in an endemic species may not be surprising only under a restrictive set of demographic assumptions (see above). Evidence for severe population size decline or extraordinarily high levels of gene flow are not borne out in the results presented here. The lack of clear genetic divergence or geographic barriers and similar levels of estimated intra and interspecific gene flow between species suggests that the gene pool of both *H. neglectus* and *H. petiolaris* is most appropriately viewed as one large collection of alleles.

A number of studies [27,33,34] have recently shown that the genomes of the more distantly related *H. petiolaris* and *H. annuus* are highly permeable to gene flow, with large portions showing very little genetic divergence in the roughly two million years since the species’ initial divergence. Nonetheless, these two species remain morphologically and ecologically divergent through the action of multiple strong reproductive barriers. It is possible that a similar phenomenon is occurring here, although it is unlikely for several reasons. While there is some degree of reproductive isolation between *H. neglectus* and *H. petiolaris* due to chromosomal differentiation and possibly some genic factors, isolating barriers between these two species are far lower than barriers between *H. petiolaris* and *H. annuus*; in fact they are comparable to barriers between different chromosome races within *H. petiolaris*. Second, while rates of introgression between *H. petiolaris* and *H. annuus* appear to be exceptionally high for two distinct species, they are still considerably lower than levels of gene flow within either species. Third, *H. neglectus* and *H. petiolaris* are far more similar to each other morphologically, ecologically, and chromosomally than are *H. petiolaris* and *H. annuus*, which differ by a minimum of 11 large-scale rearrangements [35]. And fourth, both *H. annuus* and *H. petiolaris* are very widespread and have species-wide population census sizes well into the millions of individuals, consistent with their high levels of genetic diversity and large effective population sizes. In contrast, *H. neglectus* is limited to a very small range, and while its census size is not known precisely, it is probably under our estimated effective size of 2.3 million. While it is possible under certain conditions for effective size to be larger than census size [36], those conditions are not realistic in this case; the huge amount of genetic variation in *H. neglectus* is not consistent with any substantial degree of genetic isolation.

## 3. Experimental Section

### 3.1. Sampling and Molecular Methods

**Figure 1.**
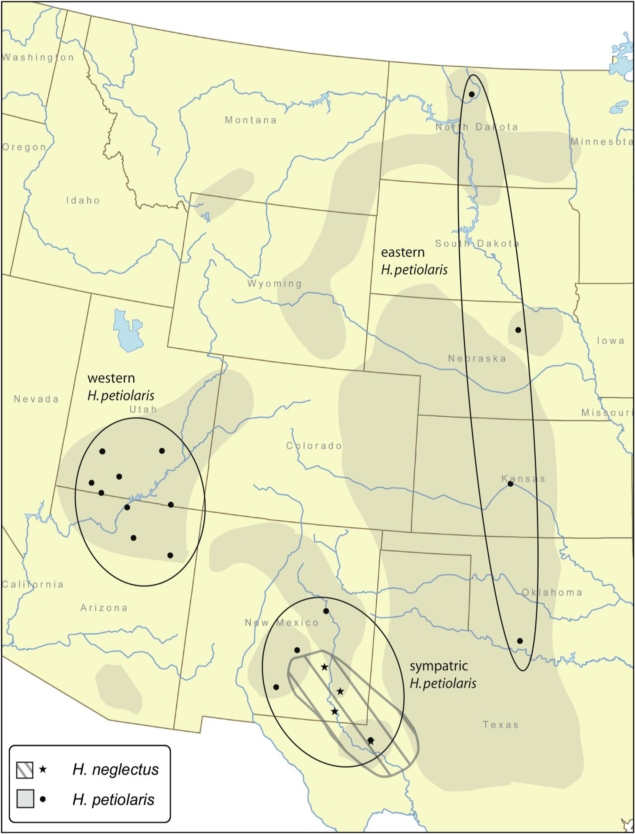
Sampling localities for *H. petiolaris* and *H. neglectus* individuals used in this study. Respective ranges are denoted with shading. Solid ovals encircle *H petiolaris* population groups used in AMOVA and MIGRATE analyses. Latitude, longitude and sample sizes are given in Table 6.

DNA samples of *H. petiolaris* were obtained from within the Rieseberg Lab from previous studies. Our sampling includes both subspecies of *H. petiolaris*, in roughly equal numbers (20 individuals from ssp. *petiolaris*, and 28 individuals from ssp. *fallax*). Achenes from *H. neglectus* were obtained from the United States Department of Agriculture Germplasm Resources Information Network and were germinated and grown in the Indiana University greenhouses. Sampling locality data for both species are given in Table 6 and Figure 1. Species identity of *H. neglectus* plants was verified morphologically by Charles Heiser (personal communication), who first described the species [13]. DNA was extracted from fresh leaf tissue using a DNeasy Plant Minikit (QIAGEN, Valencia, CA). DNA sequences from two species of the closely related genus *Bahiopsis,* formerly *Viguiera* [37], *B. lanata* and *B. reticulata*, were used as outgroups. Because most annual sunflowers have large effective population sizes, outgroups that are fairly distant genetically are required to ensure that character state is not confounded by shared ancestral polymorphism.

Nine genetic markers were chosen based on previous amplification success in a wide range of *Helianthus* species, including *H. petiolaris.* Primers for all markers were developed based on several *Helianthus* EST libraries (http://compgenomics.ucdavis.edu/) and manufactured by Integrated DNA Technologies (USA). Primer sequences and PCR amplification profile conditions are available in Strasburg and Rieseberg [18]. The nine markers are located on seven separate linkage groups, and all nine are unlinked (data not shown). Unincorporated primers and dNTPs were removed from the PCR reaction using 1 μL ExoSAP-IT (USB) per 10 μL reaction. All PCR products were quantified on a 1.5% agarose gel using a low DNA mass ladder (Invitrogen, USA).

Sequencing reactions were resolved using an ABI3730 automated sequencer (Applied Biosystems, Foster City, California, USA). The sequence results were then trimmed and aligned by eye using the computer program Sequencher (Gene Codes). Individuals with multiple sites of length heterozygosity or multiple single nucleotide polymorphisms (SNPs) were resolved using TOPO-TA cloning kits (Invitrogen, USA), otherwise individuals heterozygous for a single indel were resolved by comparing forward and reverse sequences at variable sites. All *H. neglectus* sequences presented here are new, as are most *H. petiolaris* sequences at loci JLS1040, JLS1615, and JLS1747; other *H. petiolaris* sequences and all *Bahiopsis* sequences come from a previous study [18]. All sequences have been submitted to GenBank, and accession numbers are given in Supplementary File S2.

### 3.2. Data Analysis

Coding regions and reading frames for each locus were identified by comparing *Helianthus* genomic and EST sequences with genomic and coding sequences for the closest *Arabidopsis* BLAST hit. For two loci, coding regions and reading frames could not be identified; these loci were considered to be noncoding.

Nucleotide diversity (π) using the Jukes-Cantor [38] correction and Watterson’s θ [39] were estimated using DnaSP version 4.50.3 [40]. Gross and net sequence divergence between *H. petiolaris* and *H. neglectus* were calculated in SITES [41]. Net divergence is equal to gross divergence minus average diversity within each species. The four categories of segregating sites described in Wakeley and Hey [16] were also summarized in SITES. Neighbor joining trees were constructed in PAUP 4.0b10 [42] to estimate relationships among haplotypes. AMOVAs were performed using Arlequin version 3.11 [43]. For these analyses, three regions were defined in *H. petiolaris* based on geography and breaks in sampling (see Table 6 and Figure 1). Due to the very limited geographic range of *H. neglectus*, all populations in this species were grouped together. Standard interpretations of AMOVA assume selective neutrality and random mating within populations; neither of these assumptions is likely to be violated in our data, as indicated by the results of Fu’s and Tajima’s tests (see results), and the fact that both species are obligate outcrossers with typically large population census sizes. Mantel tests of correlation between geographic and genetic distance between populations were performed in Arlequin, with 10,000 permutations performed to assess significance. Geographic distance matrices were calculated from latitude/longitude data using the program Passage (http://www.passagesoftware.net/).

To assess whether selection has affected the loci examined and also to test for past population expansion, Fu’s [17] and Tajima’s [44] tests of neutrality were performed using 1,000 simulations in Arlequin. These tests compare two different measures of genetic variation that are expected to be equal under conditions of selective neutrality and long-term demographic stability; significant differences between these two measures may be attributable to the effects of natural selection or population size changes. Fu’s test has been shown to be much more sensitive in detecting population growth than Tajima’s [17].

In addition to Fu’s and Tajima’s tests, the Hudson-Kreitman-Aguade (HKA) test [45] was used to test for neutrality. The HKA test compares the frequency of polymorphisms within species to differences between species to examine patterns of selection. This test examines the frequency of occurrence of both synonymous and non-synonymous substitutions across loci sampled. If selective constraints are the same across loci, ratios of intraspecific polymorphisms to interspecific differences should be the same across loci; in contrast, variable patterns of selection across loci may result in variation in polymorphism: divergence ratios. This test assumes selective neutrality, independence among loci, no recombination within loci, and constant population size in each species. Our data likely violate the latter two assumptions to some degree; the recombination assumption is a conservative one, but the degree to which the test is robust to population size changes is not well understood [46]. HKA tests were performed using the program HKA (available at http://lifesci.rutgers.edu/~heylab/HeylabSoftware.htm#HKA) with 10,000 coalescent simulations to assess significance.

Estimates of long-term inbreeding effective population sizes and rates of gene flow among regions were made under a Bayesian inference framework using the program MIGRATE [31,47]. Geographic regions were the same as those used in AMOVAs. Uniform priors were used for all parameters, and three independent runs were performed to verify convergence. All runs involved five independent chains, and 2.5 million steps were recorded following a burn-in of 10,000 steps, with a sampling increment of 20 steps. All effective sample sizes were at least 17,000 (most were several hundred thousand or higher). To calculate demographic quantities from MIGRATE parameter estimates, we used a per site, per year silent substitution rate of 1 × 10^−8^ (MS Barker and LH Rieseberg, unpublished manuscript), and a one year generation time. The model implemented in MIGRATE assumes no recombination within loci. We used DnaSP version 4.50.3 to infer apparently non-recombining blocks of sequences based on the algorithm of Hudson and Kaplan [48], and the largest non-recombining block was used in the MIGRATE analyses. MIGRATE also assumes independence among loci and selective neutrality, both of which are consistent with our data. We chose MIGRATE rather than another popular program for estimating effective sizes and gene flow rates, IMa [49], because the current implementation of IMa is limited to two populations, and we were interested in measuring gene flow among multiple regions within the two species. Likewise, we did not use the newest version of IMa, IMa2 [50], which allows more than two populations, because it requires that the tree topology for the populations or species being analyzed be known, which is not the case here.

## 4. Conclusions

In spite of their strong genetic similarity, *H. neglectus* and *H. petiolaris* are clearly to some extent phenotypically and ecologically distinct. In describing *H. neglectus* and comparing it to its nominal sister species, Heiser [13] considered its status as a separate species or subspecies of *H. petiolaris* to be “rather arbitrary,” because the differences between *H. neglectus* and *H. petiolaris* appear to be similar in degree and kind to the differences between different *H. petiolaris* subspecies. With the additional genetic data we have presented here, *H. neglectus*’ comparability to currently recognized *H. petiolaris* subspecies appears even stronger. While more genetic data would certainly be useful in clarifying genomic patterns of introgression and identifying any genic or structural factors that may contribute to local adaptation or reproductive isolation, based on the available data the populations currently recognized as *H. neglectus* do not appear to warrant recognition as a distinct species.

## Figures and Tables

**Table 1.** General information and summary statistics for all nine loci. π and θ values are per base pair.

				**H. petiolaris**			**H. neglectus**				
		
**Locus**	**Linkage group**	**Aligned size (bp)**	**Protein homology (based on closest Arabidopsis thaliana BLAST hit)**	**No. of seqs.**	**π (silent)**	**θ (silent)**	**No. of seqs.**	**π (silent)**	**θ (silent)**	**% Gross divergence**	**% Net divergence**
(2213)	6	688	Scarecrow transcription factor family protein	32	0.019	0.024	16	0.017	0.022	2.66	0.46
JLS244	9	571	Proton-dependent oligopeptide transport (POT) family protein	30	0.024	0.036	16	0.030	0.037	1.76	0.12
JLS720a	16	687	Cellulose synthase-related protein	32	0.028	0.037	2	0.040	0.039	1.93	0.00
JLS810R1	13	1326	Auxin efflux carrier protein family	32	0.053	0.039	16	0.063	0.058	4.43	0.37
JLS1040	10	659	Serine/threonine phosphatase	96	0.046	0.063	16	0.045	0.062	2.43	0.07
JLS1615	14	575	Unknown protein	96	0.044	0.082	16	0.051	0.060	1.14	0.06
JLS1747R2	17	789	Amino acid permease family protein	90	0.062	0.093	16	0.061	0.076	2.47	0.04
JLS1953	17	454	Galacturonosyltransferase 4	32	0.016	0.024	16	0.008	0.011	1.19	0.05
JLS2899	14	776	Unknown protein	32	0.032	0.057	16	0.052	0.067	1.48	0.04
Average		725			0.036	0.051		0.041	0.048	2.17	0.14

**Table 2.** Summary of four types of segregating sites found within or between species (see Wakeley and Hey [16]).

**Locus**	**Polymorphic in *H. petiolaris* only**	**Polymorphic in *H. neglectus* only**	**Polymorphic in both species**	**Fixed differences between species**
(2213)	33	30	15	0
JLS244	24	22	13	0
JLS720a	40	8	10	0
JLS810R1	27	44	39	0
JLS1040	79	36	37	0
JLS1615	47	8	19	0
JLS1747R2	98	29	56	0
JLS1953	32	5	9	0
JLS2899	30	28	29	0
Overall	410	210	227	0

**Table 3.** Results of Tajima’s (D) and Fu’s (Fs) tests of neutrality, with significance assessed based on 1000 simulations using Arlequin. Significant results are presented in bold; tests marked with asterisks are significant after Bonferroni correction. Tajima’s D and Fu’s Fs were not calculated for locus JLS720a in *H. neglectus*, as only two haplotypes were sampled.

	***H. petiolaris***				***H. neglectus***			
	**Tajima’s D**		**Fu’s Fs**		**Tajima’s D**		**Fu’s Fs**	
**Locus**	**D**	**p value**	**Fs**	**p value**	**D**	**p value**	**Fs**	**p value**
(2213)	−0.491	0.353	−14.547	<0.001*	−0.738	0.234	−2.918	0.052
JLS244	−0.857	0.214	−24.231	<0.001*	−0.893	0.218	−9.227	<0.001*
JLS720a	−1.008	0.166	−23.772	<0.001*	N/A	N/A	N/A	N/A
JLS810R1	1.423	0.952	−6.959	**<0.008**	0.344	0.702	−5.905	**0.006**
JLS1040	−1.279	0.070	−23.957	<0.001*	−1.196	0.118	−3.941	**0.039**
JLS1615	−1.721	**0.017**	−25.183	<0.001*	−0.932	0.196	−11.118	<0.001*
JLS1747R2	−1.209	0.105	−23.990	0.001*	−1.197	0.110	−3.922	**0.029**
JLS1953	−1.326	0.074	−24.396	<0.001*	−0.878	0.205	−6.512	0.005*
JLS2899	−1.698	**0.029**	−24.103	<0.001*	−1.014	0.161	−6.586	0.005*

**Table 4.** Effective population size estimates (modes and 95% confidence intervals) made using MIGRATE. Regions within *H. petiolaris* are shown in Figure 1. These biological quantities were calculated from MIGRATE parameter estimates as described in the methods.

**Population**	**Mode**	**2.5%**	**97.5%**
Eastern *H. petiolaris*	2.42 × 10^5^	0	4.87 × 10^5^
Western *H. petiolaris*	2.92 × 10^5^	4.74 × 10^4^	5.33 × 10^5^
Sympatric *H. petiolaris*	6.42 × 10^5^	1.51 × 10^5^	1.97 × 10^6^
*H. neglectus*	2.29 × 10^6^	8.45 × 10^5^	9.20 × 10^6^

**Table 5.** Gene flow (N_e_m) estimates made using MIGRATE. Receiving region is listed across the top of the table, and source region is listed down the left side. Values in parentheses are 95% confidence intervals. Regions within *H. petiolaris* are shown in Figure 1. EP = eastern *H. petiolaris* populations; WP = western *H. petiolaris* populations; SP = *H. petiolaris* populations sympatric with *H. neglectus*; NE = *H. neglectus* populations. These biological quantities were calculated from MIGRATE parameter estimates as described in the methods.

**Gene Flow (N_e_m)**	**into EP**	**into WP**	**into SP**	**into NE**
from EP	-	0.01 (0–3.80)	0.01 (0–4.98)	0.01 (0–5.08)
from WP	0.01 (0–3.80)	-	0.01 (0–5.50)	0.01 (0–4.83)
from SP	0.01 (0–3.88)	0.01 (0–3.78)	-	0.01 (0–5.13)
from NE	0.01 (0–3.90)	0.01 (0–3.78)	0.01 (0–4.70)	-

**Table 6.** Sampling localities. Geographic sites are shown in Figure 1. Population superscripts refer to AMOVA and MIGRATE groupings (1 = eastern *H. petiolaris* populations, 2 = western *H. petiolaris* populations, 3 = *H. petiolaris* populations sympatric with *H. neglectus*, 4 = *H. neglectus* populations).

**Species**	**Population**	**Location**	**Latitude (N)**	**Longitude (W)**	**No. of individuals**
*H. petiolaris*	KSG^1^	Great Bend, KS	38.36	98.76	5
	NDM^1^	Minot, ND	48.30	100.71	5
	NEO^1^	O’Neill, NE	42.29	98.63	5
	OK1^1^	Cotton Co., OK	34.33	98.40	5
	Pet_1283^2^	Coconino Co., AZ	36.04	111.19	3
	Pet_1287^2^	Kane Co., UT	37.24	112.86	2
	Pet_1604^2^	Kane Co., UT	37.03	112.48	2
	Pet_1277^2^	Coconino Co., AZ	36.78	111.55	2
	Pet_1279^2^	Navajo Co., AZ	35.75	109.91	2
	Pet_1292^2^	Hanksville, UT	38.35	110.69	2
	Pet_1285^2^	Kane Co., UT	37.52	111.98	1
	Pet_1323^2^	San Juan Co., UT	37.03	110.13	1
	Pet_1271^2^	Iron Co., UT	38.08	112.69	1
	Pet_1325^2^	Iron Co., UT	38.08	112.68	1
	Pet_1327^3^	Puerto de Luna, NM	34.82	104.63	3
	Pet_1332^3^	Monahans, TX	31.62	102.90	4
	Pet_lin^3^	Lincoln Co., NM	33.95	105.41	2
	Pet_trb^3^	Tularosa Basin, NM	32.90	105.96	2
*H. neglectus*	NMRO^4^	Roswell, NM	33.40	104.53	2
	NMLO^4^	Loving, NM	32.29	104.09	2
	NMLH^4^	Loco Hills, NM	32.82	103.97	2
	TXMO^4^	Monahans, TX	31.60	102.89	2
